# Site-specific prediction of *O*-GlcNAc modification in proteins using evolutionary scale model

**DOI:** 10.1371/journal.pone.0316215

**Published:** 2024-12-31

**Authors:** Ayesha Khalid, Afshan Kaleem, Wajahat Qazi, Roheena Abdullah, Mehwish Iqtedar, Shagufta Naz

**Affiliations:** 1 Department of Biotechnology, Lahore College for Women University, Lahore, Pakistan; 2 Department of Computer Science, COMSATS University, Islamabad, Pakistan; 3 Department of Zoology, Lahore College for Women University, Lahore, Pakistan; University College Dublin, IRELAND

## Abstract

Protein glycosylation, a vital post-translational modification, is pivotal in various biological processes and disease pathogenesis. Computational approaches, including protein language models and machine learning algorithms, have emerged as valuable tools for predicting *O-*GlcNAc sites, reducing experimental costs, and enhancing efficiency. However, the literature has not reported the prediction of *O*-GlcNAc sites through the evolutionary scale model (ESM). Therefore, this study employed the ESM-2 model for *O-*GlcNAc site prediction in humans. Approximately 1100 O*-*linked glycoprotein sequences retrieved from the *O-*GlcNAc database were utilized for model training. The ESM-2 model exhibited consistent improvement over epochs, achieving an accuracy of 78.30%, recall of 78.30%, precision of 61.31%, and F1-score of 68.74%. However, compared to the traditional models which show an overfitting on the same data up to 99%, ESM-2 model outperforms in terms of optimal training and testing predictions. These findings underscore the effectiveness of the ESM-2 model in accurately predicting *O-*GlcNAc sites within human proteins. Accurately predicting *O*-GlcNAc sites within human proteins can significantly advance glycoproteomic research by enhancing our understanding of protein function and disease mechanisms, aiding in developing targeted therapies, and facilitating biomarker discovery for improved diagnosis and treatment. Furthermore, future studies should focus on more diverse data types, longer protein sequence lengths, and higher computational resources to evaluate various parameters. Accurate prediction of *O*-GlcNAc sites might enhance the investigation of the site-specific functions of proteins in physiology and diseases.

## Introduction

The protein structures and functions are regulated in a biochemical process of glycosylation where sugar molecules covalently bond with proteins or lipids, commonly involving amino acids like asparagine (N, Asn), tryptophan (W, Trp), serine (S, Ser), and threonine (T, Thr) [1]. Biological functions such as cell adhesion, stability, protein folding, and molecular movement are affected by the crucial modification of glycosylation, eventually altering signaling processes, such as immune responses, antigenicity, and cell death by binding to the receptors within the cells [2–5].

Glycosylphosphatidylinositol (GPI) anchor attachment, *N*-linked glycosylation, *O-*linked glycosylation, and *C*-mannosylation are different types of glycosylation in which the sugar molecules and the acceptor residues in protein or lipids make specific chemical linkages [6]. The *N*-linked glycosylation involves the binding within specific sequence motifs N-X-C or N-X-T/S of the glycans to the amide nitrogen of Asn [7]. Besides, *O-*linked glycosylation involves Ser or Thr hydroxyl group, often near a Pro residue [8]. Particularly, phosphorylated Thr/Ser residues get inclined towards *O-*linked β-N-acetylglucosamine (*O-*GlcNAc) glycans, indicating that these modifications may participate through an intricate interplay [9].

The accuracy of the prediction of O-GlcNAcylation sites is of significant importance as this modification is involved in various vital pathways. While the consensus site for *N*-glycosylation is widely recognized as C/T/N-X-S, with X representing any residue except proline, the distinct *O*-GlcNAcylation pattern has not yet been identified. Hence, the development of effective prediction tools poses a significant challenge. This should be strengthened by directly linking these challenges to the specific contributions of this study. Several prediction tools, such as YinOYang (https://services.healthtech.dtu.dk/services/YinOYang-1.2/), OGTSite [10], and *O-*GlcNAcPred-II [11], are already accessible and have shown good prediction results, with specificity and sensitivity up to 95.91% and 81.05%. These models implement Neural Networks, Random Forests, or Principal Component Analysis (PCA) algorithms built on sequence data. However, these predictions depend on test data and are disparate for each tool, making it difficult to compare the results [10–12]. Furthermore, certain limitations are reported in models, such as unavailable large datasets of YinOYang experimental *O-*GlcNAcylation sites and the unbalanced ratio of negative and positive samples of *O-*GlcNAcScan [13]. This signifies a notable limitation for previously identified models.

The protein-language models (PLM) have outperformed the supervised learning models in terms of accuracy. Notably, the highest performance is attained by the evolutionary scale model (ESM) [14]. The ESM, a pre-trained model, is reported to predict the glycan bindings and various proteins, such as structure stability or function [15]. Advanced deep learning models, such as transformer-based models, have also been utilized to identify relationships and patterns within protein sequences [16].

*O-*GlcNAcylation, occurring on numerous proteins across various pathways, is crucial in maintaining cellular homeostasis. The *O-*GlcNAc cycling dysregulation has been implicated in various pathologies such as Alzheimer’s disease, cancers, diabetes, and intellectual disabilities associated with *O-*GlcNAc transferase congenital disorder of glycosylation (OGT-CDG). Modifying proteins through *O-*GlcNAc transferase (OGT) is crucial for developing the nervous system and the functioning of neurons. However, when there are missense mutations in OGT, it can result in severe neurological disorders [17,18]. Furthermore, *O-*GlcNAcylation influences several cellular processes, including autophagy, apoptosis, and protein degradation, as well as governing protein biogenesis, transcription, translation, and stability [19]. Therefore, accurate prediction of *O-*GlcNAcylation sites is crucial for understanding its biological importance and potential therapeutic applications due to its significant role in various diseases and cellular functions.

Understanding the functional roles of these proteins in disease is costly to predict experimentally [20,21]. Therefore, computational methods are employed for cost-effective predictions. Databases, such as *O-*GlcNAc (https://www.oglcnac.mcw.edu/) v1.3 provide comprehensive human *O-*GlcNAcylated proteins information, including data on their *O-*GlcNAc sites, corresponding references, and identification methods [22,23].

In this study, the objective was to predict *O-*GlcNAc sites in mammalian proteins using machine learning algorithms. The protein language model ESM was employed to overcome the limitations of previous models, such as unbalanced negative and positive sample ratios. The model, based on transformers, is reported for site-specific prediction. The ESM-2 family of models is the largest protein language model to date, with significantly improved performance over previous models. The ESM-2, with 150M parameters, employs an attention mechanism to effectively learn the interaction patterns between pairs of amino acids in the input sequence, addressing the limitation of undefined O-GlcNAcylation patterns by interpreting protein sequences as tokens or characters [24].

Proteins with predicted *O-*GlcNAc sites were used to train the model, followed by an evaluation of the performance of the model in predicting new sites. Notably, the ESM, transformer-based model, has not been reported to predict the *O-*GlcNAc sites in mammalian proteins. Therefore, this study aimed to assess the effectiveness of the ESM in accurately identifying *O-*GlcNAc sites, thereby developing the computational techniques for glycoproteomic research.

## Methodology

### Study design

The study aims to develop a predictive model utilizing ESM token classification to predict the *O-*GlcNAc sites in mammalian protein sequences.

### ESM analysis

The study employed ESM-2 model that provides 480 output dimensions after embedding. The ESM model effectively captured the crucial characteristics, which are trained on extensive and evolutionary information of the protein sequences. The model depicted various structural and functional characteristics by giving protein sequences as input, which generated a vector of numerical features [25]. Notably, previous models to predict *O*-GlcNAc sites employed larger batch sizes (128) and less optimized learning rates, potentially leading to increased optimization difficulties and memory management issues [26]. Conversely, in this study, the fit method of TensorFlow was employed to specify the number of epochs for controlled training iterations. Moreover, higher computational resources are required for larger batch sizes, resulting in optimization difficulties [27]. Similarly, optimizing the learning rate schedule to attain the maximum possible learning rate without inducing training instabilities in limited computer resources. Therefore, the hyperparameters selected for the training of the ESM-2 model include a batch size of 8, a learning rate of 1e-5, and 10 epochs with gradient accumulation steps set to 16, assuring that the model fits within the available GPU or CPU memory. Furthermore, the mentioned hyperparameters prevented memory outage errors and provided an equilibrium between computational efficiency and the stability of gradient updates, leading to continuous and comprehensive protein sequence convergence throughout the training with respect to available memory and computational resources.

This study should also benefit from a brief discussion on the choice of hyperparameters for training the ESM-2 model, including the rationale behind the chosen batch size, learning rate, and epochs. Also explain the novelty of your work and how it is better than already existing tools.

### Dataset collection and preprocessing

The *O-*GlcNAc database was utilized as the source for the retrieval of the *O-*linked glycoproteins primary dataset [25]. The sequence of the glycoproteins can comprise the *O-*GlcNAc sites at any Ser or Thr amino acid [28]. Protein sequences are substantially longer than text sequences, increasing computational complexity. Thus, high-performance computing facilities may find it difficult to employ ESM-2 due to the high number of parameters and computational complexity [29]. Consequently, data filtration was performed to proceed with analysis with only site-specific protein sequences. Hence, the rows without the *O*-GlcNAc sites were eliminated. Subsequently, excluding null value rows and performing data trimming, the remaining *O*-linked glycoproteins and their respective sequences were included for ESM analysis. Moreover, considering the computational constraints and resources such as Google Colab and T4 GPU, the minimum length of 500 and maximum length of 800 amino acids were selected to trim the protein sequences. The ESM model is reported to train over 1022 amino acids and cannot be extended during the test time [30].

The data in this study is available for reproducibility purposes at https://www.kaggle.com/datasets/esm2oglcnac/o-glcnac-prediction-model

### Feature encoding

Following data preprocessing, the essential libraries, including ‘os,’ ‘transformers,’ ‘pandas,’ ‘torch,’ ‘numpy,’ ‘random,’ and ‘sklearnmetrics’ were imported. Tokenization, encoding, and training were facilitated using the ‘EsmTokenizer’ and ‘EsmForTokenClassification’ classes from the ‘transformers’ library, along with ‘Trainer’ and ‘TrainingArguments’ for model training. The selected protein sequences underwent feature encoding. Subsequently, the preprocessed data, stored initially in *csv* file format, was transformed into the Parquet format for data training. Tokenization of protein sequences was performed using the EsmTokenizer, with each sequence tokenized based on the sequence length, with a maximum length of 800 tokens. Each amino acid within the sequences was tokenized by two letters: ’N’ for all other amino acids and ’G’ for Ser and Thr.

### Model training and evaluation metrics

The tokenized datasets were converted into a suitable format using the custom preprocessing function for model training. The training process was initiated using the Trainer class, configured with training arguments specifying the learning rate, batch size, number of epochs, and other training parameters. The AdamW optimizer and linear learning rate scheduler were utilized for efficient training.

During training, model performance was evaluated on the validation dataset after each epoch. Evaluation metrics such as accuracy, the ratio of correctly predicted instances over the total number of instances; precision, the ratio of correctly predicted true positive predictions out of all positive predictions; recall, the ratio of correctly predicted true positives out of all actual positives; and F1-score, a harmonic mean of recall and precision, were calculated using a custom function [31].

The confusion matrix is utilized to analyze the performance of the model and prediction accuracy. The matrix is classified into rows and columns, where rows show the true labels and columns represent the predicted labels [32]. Similarly, this model elucidates the accurate and inaccurate occurrences per class by comparing predicted and actual labels [33]. Moreover, based on the evaluation of the performance metrics of the model, the estimated counts of true negatives (TN), true positives (TP), false negatives (FN), and false positives (FP) were calculated using the provided total number of samples (N = 1113) and the accuracy, precision, and recall of the model [34].

### Prediction and testing

The trained model was utilized to predict *O-*GlcNAc sites on the test dataset. The protein sequences were tokenized, and site predictions were generated using the trained model. Standard performance metrics were implemented to evaluate the predictions, and the results were analyzed to assess the effectiveness of the model in predicting *O-*GlcNAc sites.

### Comparison with traditional models

To assess the performance of the language-based model developed in this study compared to the traditional ML-based models, we employed Moreover, to assess the model performance compared to the traditional ML algorithms, we employed AdaBoost (ADA), Decision Trees (DT), Extra Trees (ET), K-Nearest Neighbors (KNN), LightGBM (LGBM), Logistic Regression (LR), Multi-Layer Perceptron (MLP), Naive Bayes (NB), Random Forest (RF), Support Vector Machine (SVM), and XGBoost (XGB), ensuring a comprehensive evaluation of various machine learning algorithms to identify the best-performing model.

## Results

### Dataset characteristics

The primary dataset comprised approximately 9000 *O-*linked glycoproteins along with data features, such as UniProtKB IDs, entry name, organism, protein full name, oglcnacscore, oglcnac sites, phosphorylation sites, PMIDs, sequences, and amino acid count. Following the exclusion of null value oglcnac sites rows and data trimming on the length of the sequence ranging from 500 to 800 amino acids, approximately 1100 *O*-linked glycoproteins sequences and their respective *O-*GlcNAc sites were selected for data tokenization and training.

### Model development and performance evaluation

The dataset underwent preprocessing steps, including feature encoding to represent amino acids. This encoding involved labeling non-glycosylated residues as ’N’ and Ser and Thr residues with the symbol ’G.’ The *O-*GlcNAc site prediction was performed using the Facebook ESM-2.0 model with the ’Facebook/esm2_t12_35M_UR50D’ tokenizer. Subsequently, the dataset was divided into training, testing, and validation subsets using the PyArrow Parquet format.

The ESM model was fine-tuned for *O-*GlcNAc site prediction using the training dataset. The training was performed with specific hyperparameters, including a warm-up ratio of 0.1, a learning rate of 1e-5, and a batch size of 8. Additionally, the model was trained for 10 epochs with gradient accumulation steps set to 16.

**Fig 1:**
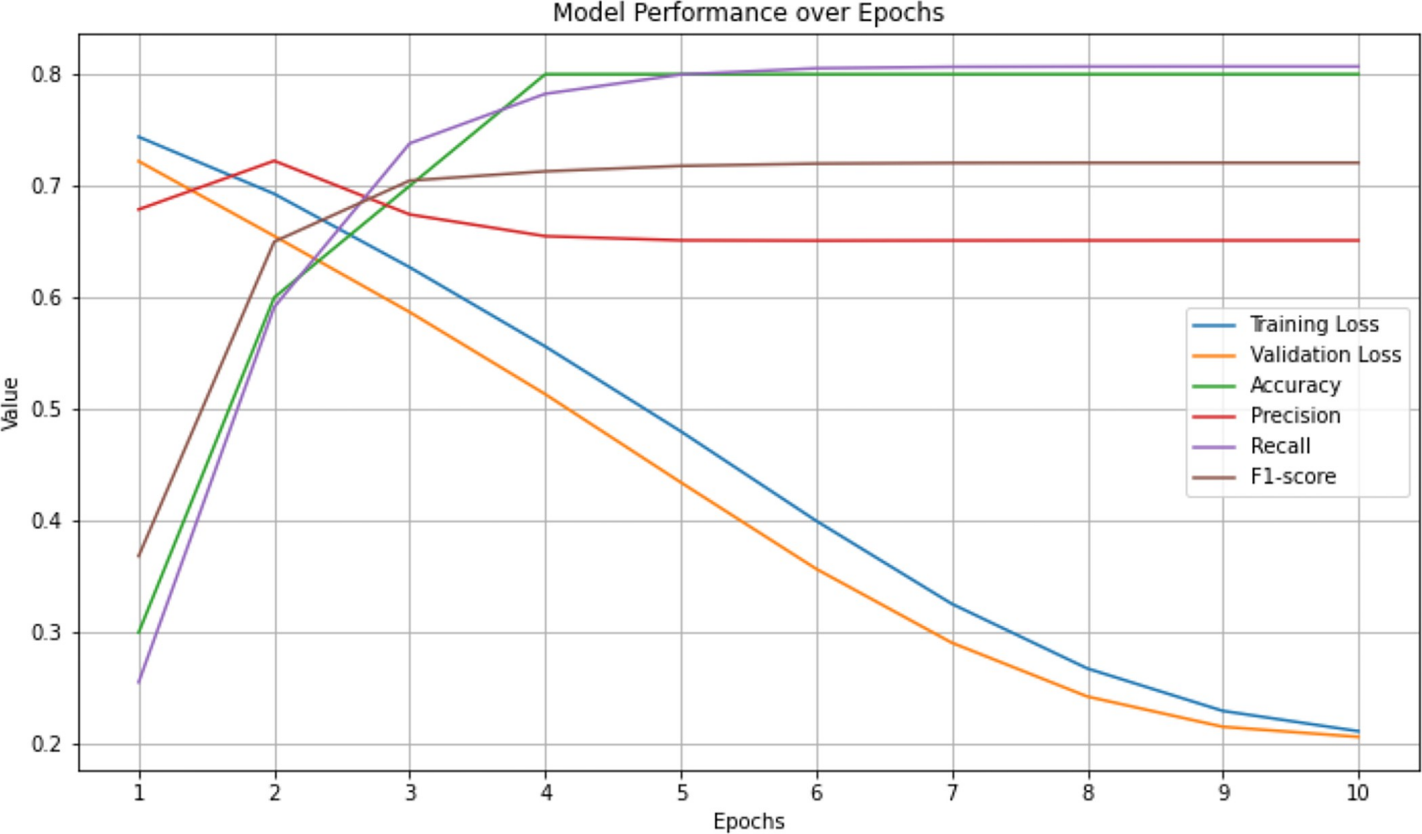
Model performance over epochs.

The trained model performance was evaluated on the validation dataset after each epoch. Initially, the model exhibited relatively high training and validation losses of 0.7439 and 0.7221, respectively, along with low accuracy, precision, recall, and F1-score values around 0.25. However, significant improvements were observed in all metrics. The model had notably reduced both training and validation losses to 0.6271 and 0.5869, respectively, while achieving higher accuracy, precision, recall, and F1-score values, surpassing 0.73 in accuracy and recall in the third epoch. The performance of the model gradually improved over subsequent epochs, reaching its highest performance in the sixth epoch with an accuracy of 0.805 and an F1-score of 0.720. Notably, from the seventh epoch, the performance of the model depicted consistency, maintaining accuracy, precision, recall, and F1-score values around 0.80 and 0.72, respectively, indicating stable convergence of the training process. The iterative training process resulted in significant improvements in model performance. The epochs, along with validation metrics, are listed in Table 1. The ESM-2 performance over epochs is illustrated in Fig 1.

**Table 1:** Training and validation metrics for *O-*GlcNAc site prediction through ESM-2.

Epoch	Training Loss	Validation Loss	Accuracy	Precision	Recall	F1
1	0.7439	0.722084	0.255425	0.67889	0.255425	0.368449
2	0.6929	0.655114	0.591408	0.722454	0.591408	0.65
3	0.6271	0.586941	0.738062	0.674416	0.738062	0.704732
4	0.5562	0.513472	0.782446	0.654959	0.782446	0.713033
5	0.4802	0.43448	0.799719	0.65123	0.799719	0.717876
6	0.4	0.35696	0.805346	0.650957	0.805346	0.719963
7	0.3257	0.290856	0.806604	0.651084	0.806604	0.720548
8	0.2678	0.242677	0.806862	0.651125	0.806862	0.720676
9	0.2298	0.215548	0.806929	0.651135	0.806929	0.720709
10	0.2116	0.206516	0.806929	0.651135	0.806929	0.720709

**Fig 2:**
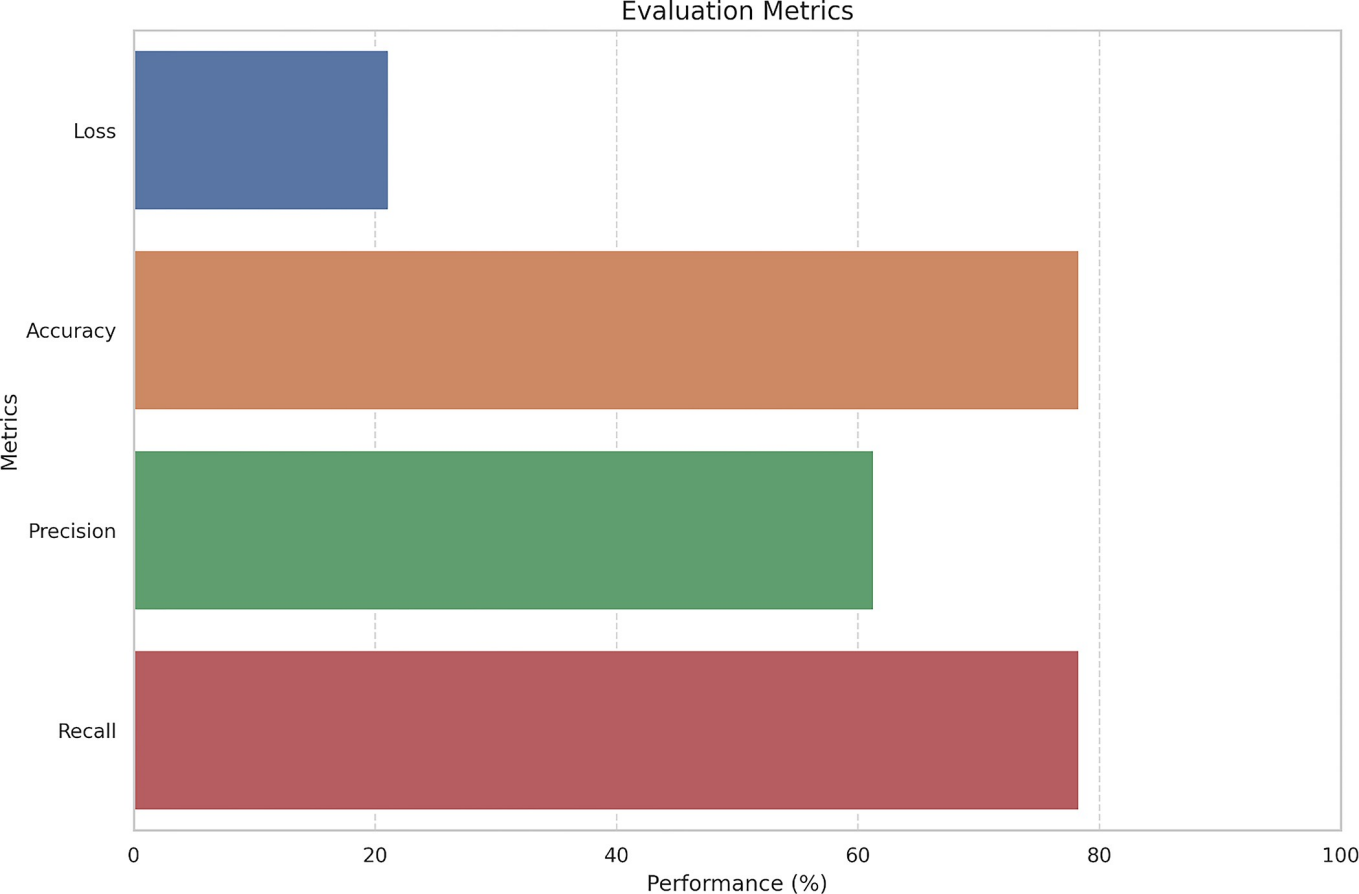
A bar chart illustration of evaluation metrics on test data.

Following the training, the model was utilized to predict *O-*GlcNAc sites on the testing dataset. The model performance was evaluated using various metrics on the test dataset. The test loss of the model was 0.21. The model attained an accuracy of 78.30 and a precision of 61.31. The recall score, reflecting the ratio correctly predicted true positives, matched the accuracy at 78.30. Additionally, the F1 score, which balances precision and recall, was computed to be 68.74. The runtime for testing was 4.64 seconds, with an average of 23.92 samples processed per second and 3.02 steps executed per second. These results collectively demonstrate the effectiveness of the model in predicting *O-*GlcNAc sites. The evaluation metrics on test data are listed and illustrated in Table 2 and Fig 2, respectively.

**Table 2:** Evaluation metrics on test data.

Matric	Value
Test Loss	0.211
Test Accuracy	0.783
Test Precision	0.613
Test Recall	0.783
Test F1-score	0.687
Test Runtime	4.641
Test Samples per Second	23.916
Test Steps per Second	3.016

**Fig 3:**
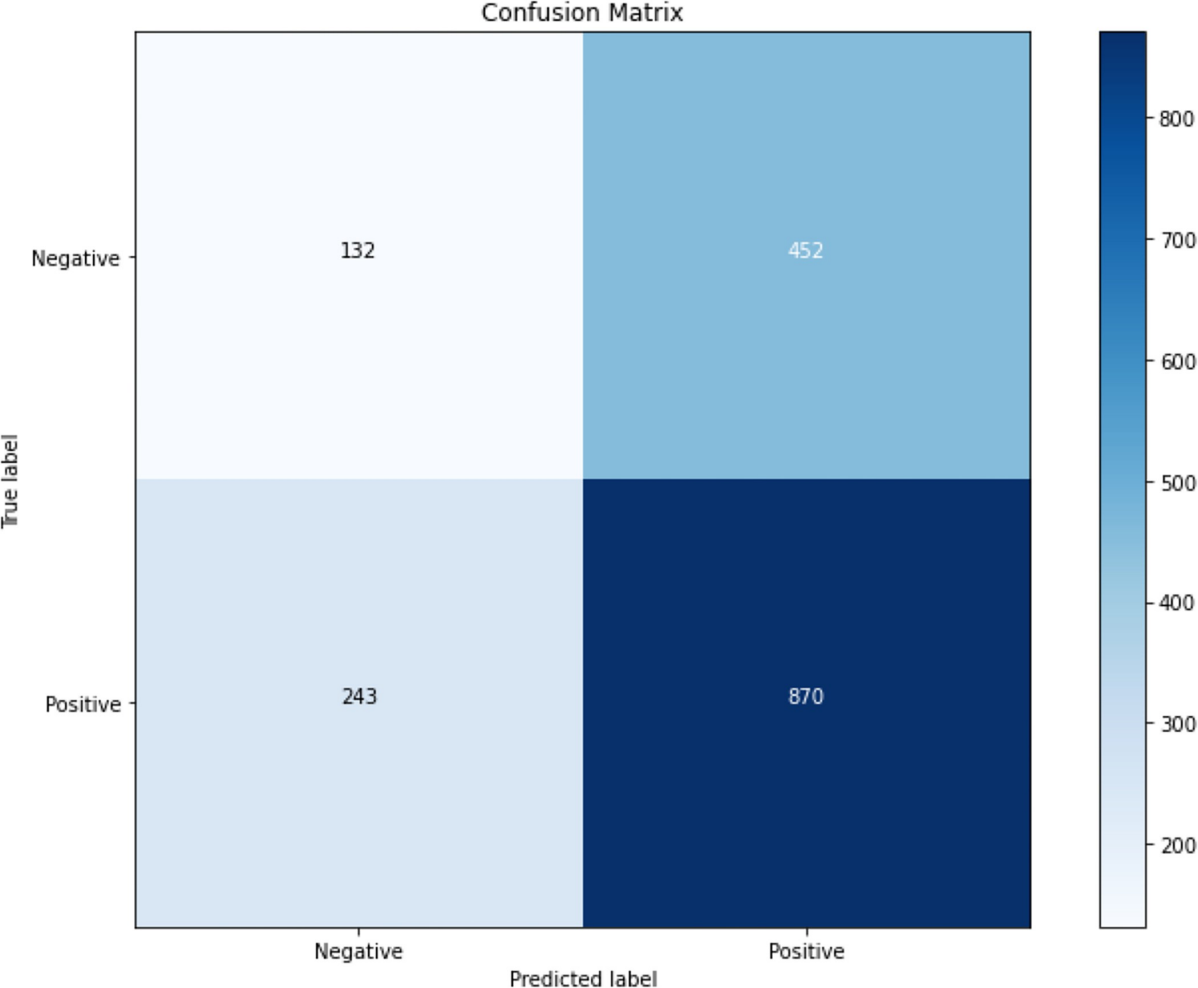
Confusion matrix representing the performance of ESM-2.

Furthermore, the estimated counts of TN, TP, FN, and FP were calculated using the provided total number of samples (N = 1113) and the accuracy, precision, and recall of the model. The model achieved a TP count of approximately 870. Conversely, an estimated FP count of approximately 452 was predicted. The TN and FN count of approximately 132 and 243 were predicted, respectively. The model accurately predicted O-GlcNAc modification sites, with a high TP count indicating its effectiveness in identifying true sites. However, it also over-predicted modifications, leading to false outputs. The model also correctly predicted non-modified sites, but a low TN count suggests it struggles to identify them. Similarly, a high FN count indicates a number of true sites are missed. However, the performance of the model can be improved by enhancing its ability to identify non-modified sites through techniques like better feature selection, addressing class imbalance, and incorporating additional biological information. These improvements would enhance the efficiency of subsequent experimental investigations and provide more comprehensive biological insights. The confusion matrix is depicted in Fig 3.

Moreover, to assess the model performance compared to the traditional ML algorithms, we employed ADA, DT, ET, KNN, LGBM, LR, MLP, NB, PLS, RF, SVM and XGB which revealed overfitting results of the models, except for NB which had an accuracy of 0.78, whereas rest of the algorithms showed > 0.99 accuracy indicating a large bias in the model training due to scarcity of the available data. However, the ESM-2 model developed in this study provides an optimal balance between overfitting and underfitting the data for development of the model while providing reasonably accurate predictions compared to the traditional ML models. Comparative visualizations for the benchmarks are given in the S1–S3 Figs. Such overfitting of the model typically is a symptom of having low amounts of data for training, which is why language-based models may work better in such cases.

The article would benefit from a discussion on the implications of the observed false positive and false negative rates, especially in the context of practical applications.

## Discussion

Protein glycosylation, a crucial process involving glycan addition to proteins, plays a crucial role in various biological processes, impacting health and disease [35]. Researchers employ computational techniques and machine learning algorithms to improve *O-*GlcNAc site prediction with reduced experimental costs [36]. Machine learning models such as support vector machine (SVM), neural net(work)s (NN), and some versions of hidden Markov models (HMM) are primarily employed in glycoproteomic [37]. However, most of these methods have manually curated their input features for prediction. The traditional machine learning models take considerable time and require technical expertise compared to transformer models, which require a significant GPU but are user-friendly and efficient [38]. Expanding on how this model can be integrated into existing workflows or its scalability would be advantageous.

Protein language models caused a recent surge in unsupervised variant effect predictors. These models use large-scale protein sequence corpora like BFD. UniRef, Pfam, and deep-learning advances in natural language processing, such as transformers, to learn evolutionary information across families of homologous proteins, unlike unsupervised models [39]. Since then, transformer-based PLMs have been developed. Self-supervised pretraining of PLMs has shown informative results in evolutionary, structural, and functional contexts, leading to variant effect prediction, supervised few-shot protein engineering, protein design and protein function prediction [40–43]. This suggests that transformer-based embedding from PLMs performs better than physicochemical, evolutionary, MSA or structural-based peptide sequence features. Recently, PLMs have gained significant attention for studying protein *O*-GlcNAc sites, and their application is expected to become increasingly crucial in glycoproteomic [44,45].

Advancements in natural language processing have led to the establishment of protein language models that learn protein representations using unlabeled protein sequence databases, varying in transformer architectures and datasets [24]. Amongst the six pre-trained PLMs (ESM, ProtXLNet, ProtBERT, ProtBERT-BFD, ProtALBERT, and TAPE) evaluated, ESM has shown the best performance with an accuracy of approximately 96% [14]. The ESM has outperformed due to its transformer architecture using six hundred and fifty million parameters, 33 layers, and extensive pre-training on UniRef50 [46]. Moreover, the ESM is reported to be accurate in protein feature extraction [47].

Multiple studies have employed the ESM-2 model for protein predictions, such as protein localization and fold prediction [48–50]. However, the application of this model for *O-*GlcNAc site prediction in humans has not been previously reported. These modifications are essential in humans, as a single monomer (*O*-GlcNAc) functionalizes the intracellular proteins. Apart from the other types of glycosylation, this *O*-GlcNAcmonomer does not extend into large polysaccharide chains and impacts the various cellular processes, contributing to health and disease. Thereby, the pre-trained protein language model (ESM-2) could potentially identify these *O*-GlcNAc sites across different species, especially in mammals [51]. Thus, this study employs the ESM-2 model for *O-*GlcNAc site prediction in humans. The preprocessing of the dataset used in this study involved protein sequence length-based data trimming and missing *O-*GlcNAc site entries exclusion to train and evaluate the dataset with ensured quality. However, another major benefit of using language-based models is that they can be trained on less amount of data without causing overfitting of the model, which we also present in this present study.

The equivalent magnetic network (EMN) fluctuating performance revealed that protein length determines the accuracy of the prediction of the *O-*GlcNAc site. Particularly, a recent study conducted by Hou (2023) that used EMN by employing ESM showed better model performance for longer protein sequences [47]. However, an accuracy of 78.30%, recall of 78.30%, a precision of 61.31%, and an F1-score of 68.74% resulted from observing the ESM-2 performance across epochs, validating the model efficacy through these performance metrics for predicting the *O-*GlcNAc sites.

Altogether, this study employed the prediction of the *O-*GlcNAc sites accurately in human proteins, exhibited by utilizing an effective pre-trained protein language model ESM-2, which evaluated the site predictions by being trained on protein sequences. Importantly, high precision, recall, accuracy, and F1-score were achieved by good performance of the model over epochs, highlighting the potential of ESM-2 in glycoproteomic research. While the ESM-2 model exhibits promising results for predicting *O*-GlcNAc sites, it is important to address the limitations in its applicability to other types of glycosylation or proteins from diverse organisms. Glycosylation patterns can differ across different glycoproteins and species, potentially influencing the performance of the model. However, the trained model employed to predict the *O*-GlcNAc sites might have the potential to predict these sites across mammals.

This study accurately predicts the *O-*GlcNAc modification sites in human proteins, where ESM-2 could be considered an efficient model. A good model performance across epochs resulted in high accuracy and precision by utilizing approximately 1100 protein sequences of the trained dataset, assisting the post-translational glycoproteomics research through the effective predictions of this model. Nevertheless, the length of the protein sequences and the unavailability of the data are the limiting factors with respect to computational resources. However, compared to the traditional models, the model based on ESM-2 for prediction of *O*-GlcNAc outperforms. Consequently, higher computational resources can train the model on larger protein sequences and datasets for better outcomes. Lastly, the model has demonstrated promising results in site-specific prediction. Hence, the model can be employed to predict *O*-GlcNAc sites across various species, particularly mammals, with the potential for broader applicability in further studies.

Nevertheless, the length of the protein sequences and the unavailability of the data are the limiting factors with respect to computational resources. However, compared to the traditional models, the model based on ESM-2 for prediction of *O*-GlcNAc outperforms. Consequently, higher computational resources can train the model on larger protein sequences and datasets for better outcomes. Lastly, the model has demonstrated promising results in site-specific prediction. Hence, the model can be employed to predict *O*-GlcNAc sites across various species, particularly mammals, with the potential for broader applicability in further studies.

## Supporting information

S1 Fig Model accuracy across the developed machine learning algorithms. (DOCX)

S2 Fig Model recall across the developed machine learning algorithms. (DOCX)

S3 Fig Model precision across the developed machine learning algorithms. (DOCX)
