# Supplementary figures and images for: Site-specific prediction of O-GlcNAc modification in proteins using evolutionary scale model
Source: PLoS One. 2024 Dec 31;19(12):e0316215. doi: 10.1371/journal.pone.0316215 (PMC11687694; doi:10.1371/journal.pone.0316215)

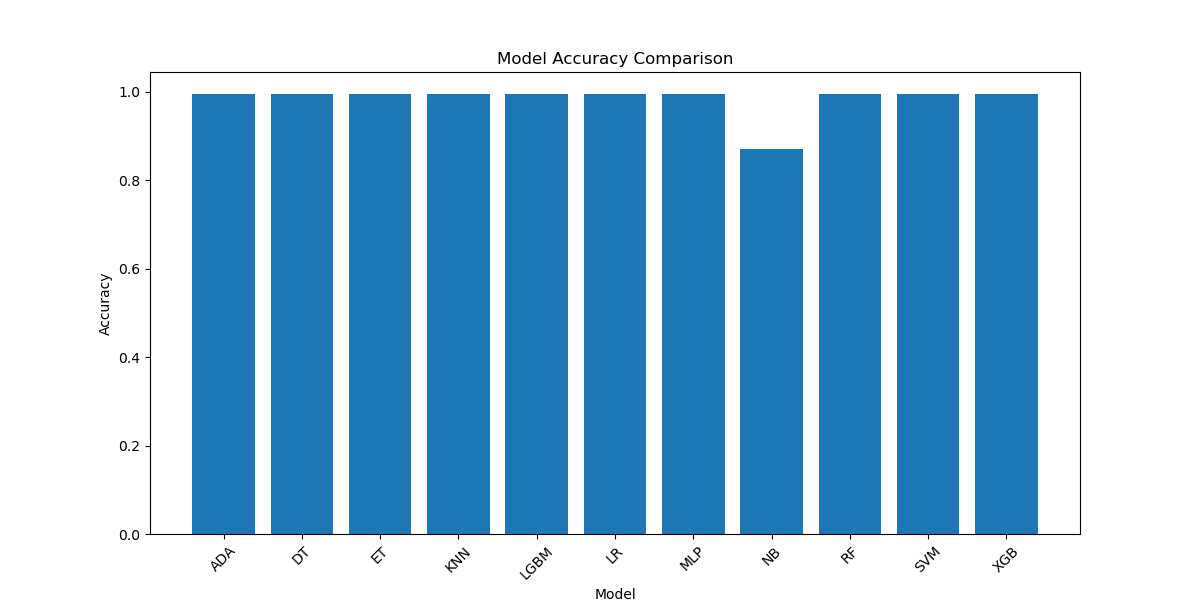


**Fig S1. Model accuracy across the developed machine learning algorithms**

Supplement: S1 Fig — (DOCX) [file pone.0316215.s001.docx]

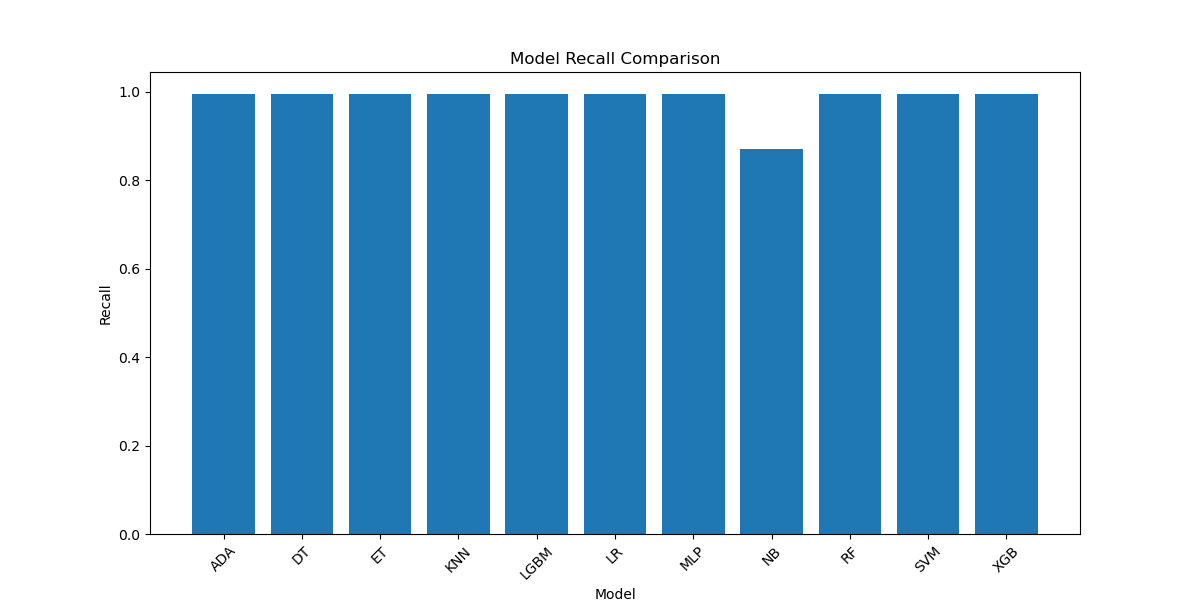


**Fig S2. Model recall across the developed machine learning algorithms**

Supplement: S2 Fig — (DOCX) [file pone.0316215.s002.docx]

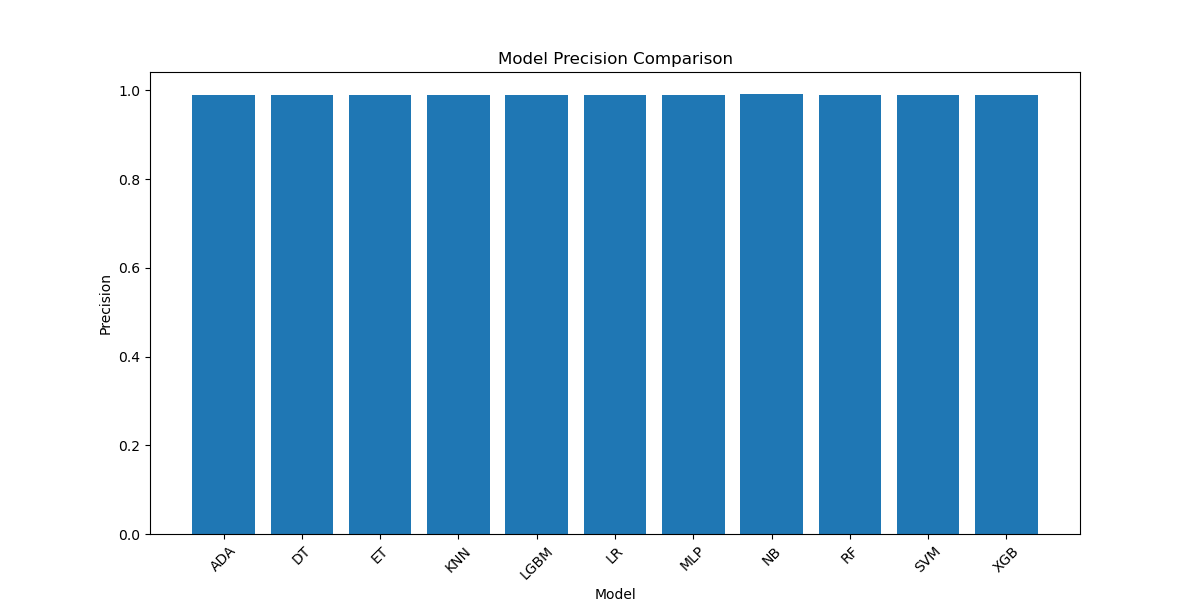


**Fig S3. Model precision across the developed machine learning algorithms**

Supplement: S3 Fig — (DOCX) [file pone.0316215.s003.docx]
